# Modified Shoelace Closure With Kirschner Wires After Forearm Fasciotomy for Rattlesnake Bite-Related Compartment Syndrome in a Pediatric Patient: A Case Report

**DOI:** 10.7759/cureus.107096

**Published:** 2026-04-15

**Authors:** Mario Hernandez Mancillas, Erick M Hernández-Mancillas, Julio C González Lara, Diego J Hernandez Camacho, Omar G Dorado Hernandez

**Affiliations:** 1 General Surgery, Hospital General Zacatecas ISSSTE, Zacatecas, MEX; 2 Surgery, Universidad Autónoma de Durango, Durango, MEX; 3 Surgery, Hospital General Zacatecas ISSSTE, Zacatecas, MEX; 4 Medical School, Hospital General del ISSSTE Zacatecas, Zacatecas, MEX

**Keywords:** acute compartment syndrome, delayed fasciotomy closure, dermatotraction wound closure, forearm fasciotomy technique, kirschner wire fixation, low cost wound closure, pediatric upper limb injury, rattlesnake bite envenomation, shoelace closure technique, snakebite compartment syndrome

## Abstract

Snakebite envenomation may cause severe local tissue injury; although compartment syndrome is uncommon, it represents a surgical emergency when present. We report the case of an 11-year-old male who presented four hours after a rattlesnake bite to the left hand with pallor, delayed capillary refill, decreased distal temperature, diminished pulses, and paresthesias. Emergency fasciotomy of the forearm and hand was performed, revealing marked edema under tension with preserved muscle viability. Seven days later, delayed closure was achieved using a modified shoelace dermatotraction technique with 1.5 mm Kirschner wires, polyester 0 sutures, and silicone protectors fashioned from Nelaton catheter segments. Progressive closure was completed over 72 hours without the need for skin grafting or negative pressure wound therapy. The patient had an uneventful recovery, and at eight-month follow-up, he demonstrated preserved range of motion, intact sensation, strength graded at 4/5, and satisfactory scar evolution. This case shows that an established dermatotraction technique can be adapted to available resources to achieve effective fasciotomy closure in selected patients.

## Introduction

Snakebite envenomation remains a significant global health problem, particularly in rural and resource-limited settings, where it is associated with considerable morbidity and mortality [1]. Local tissue effects may include edema, hemorrhage, and necrosis, occasionally progressing to acute compartment syndrome [2].

Compartment syndrome secondary to snakebite is uncommon, and its diagnosis remains controversial, as venom-induced edema may mimic elevated compartment pressures [3]. However, in patients with progressive neurovascular compromise, early recognition and surgical decompression are essential to prevent irreversible damage [4].

Wound closure following fasciotomy represents a reconstructive challenge due to persistent edema and soft tissue tension. Traditional management includes delayed primary closure, negative pressure wound therapy, or split-thickness skin grafting, each associated with specific limitations [5].

Dermatotraction techniques, such as the shoelace method, have been widely described as effective alternatives for gradual wound closure, utilizing the viscoelastic properties of the skin [6]. In this report, we describe the use of a modified shoelace technique employing Kirschner wires and silicone protectors for fasciotomy closure in a pediatric patient.

## Case presentation

**Figure 1:**
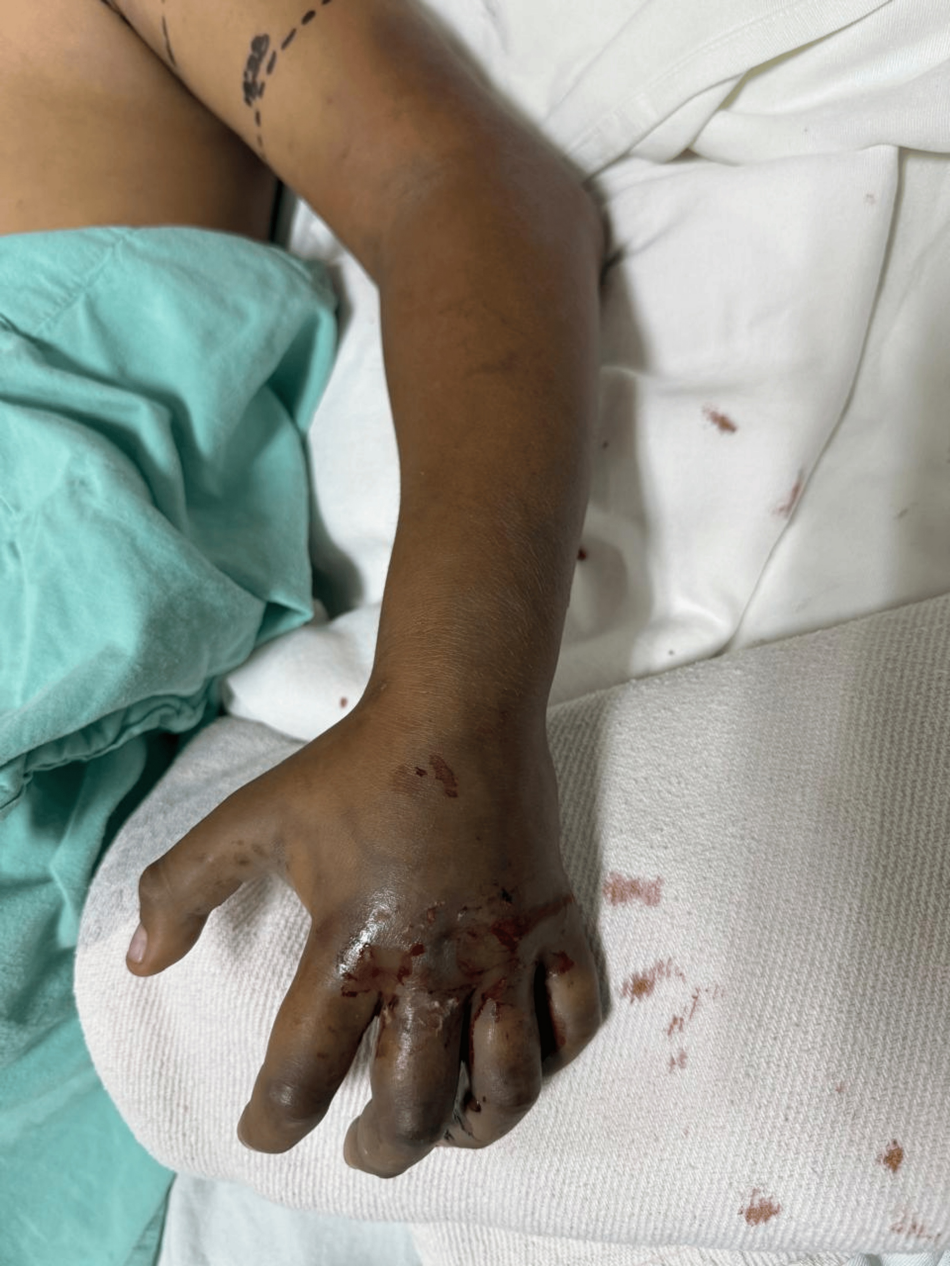
Initial presentation of the left hand after a rattlesnake bite Diffuse edema, pallor, and soft tissue compromise of the left hand are observed.

An 11-year-old male presented four hours after a rattlesnake bite to the left hand. On admission, he was hemodynamically stable but somnolent, with a Glasgow Coma Scale score of 13. Physical examination revealed diffuse edema of the left hand and distal forearm, pallor, delayed capillary refill, decreased distal temperature, diminished radial pulses, paresthesias, and rigidity of the hand in extension. The pre-surgical laboratory results showed no significant abnormalities. Figure 1 shows the initial clinical appearance of the left hand and forearm at presentation.

**Figure 2:**
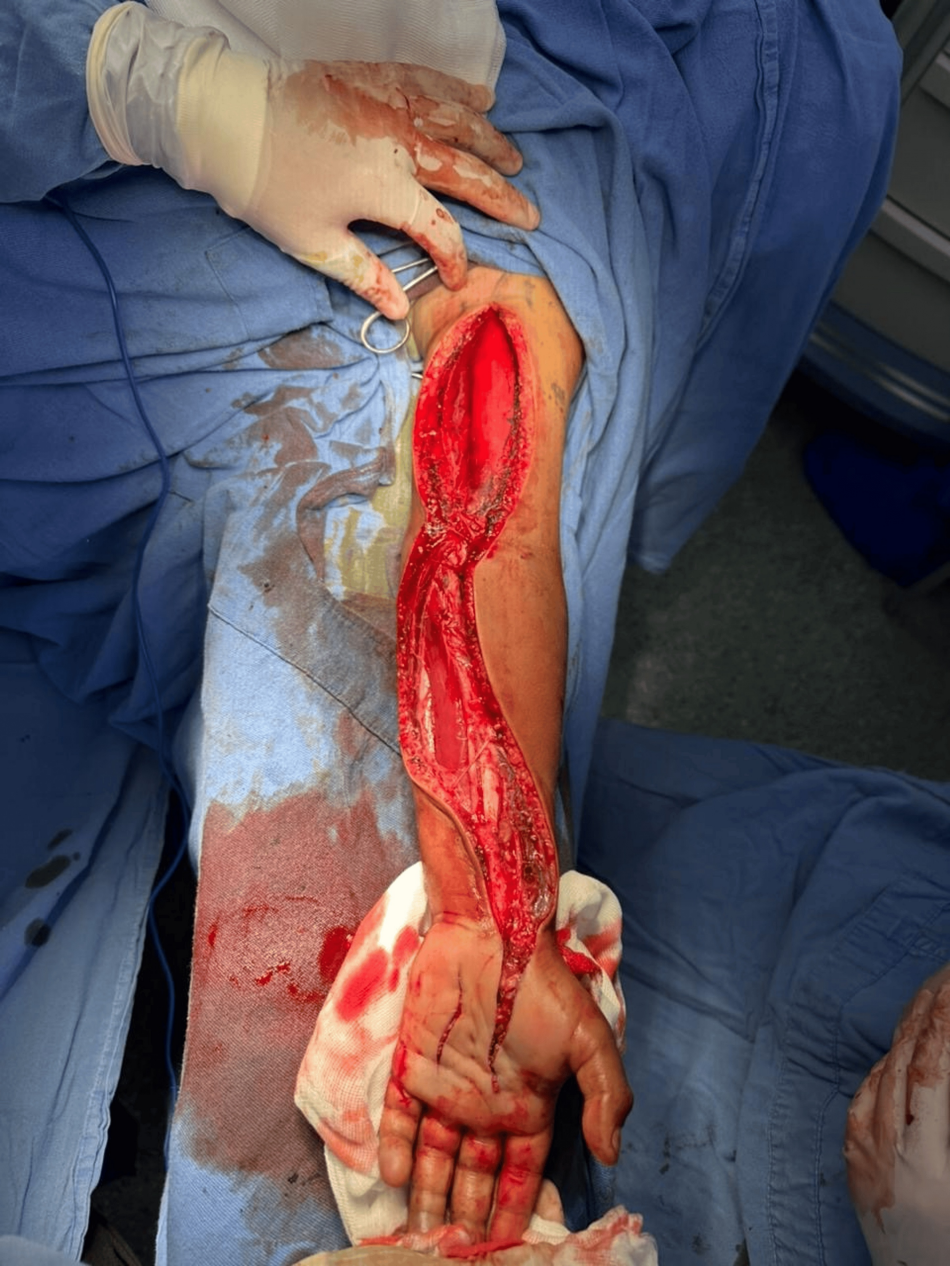
Intraoperative forearm fasciotomy Marked edema under tension with preserved muscle viability.

Ten vials of polyvalent antivenom were administered one hour before surgery. Because of progressive neurovascular compromise, the patient was taken to the operating room for urgent decompression. An S-shaped volar forearm fasciotomy and two dorsal hand incisions were performed. Intraoperatively, marked edema under tension was identified, with preserved muscle viability and no necrosis. Figure 2 shows the intraoperative appearance after fasciotomy.

**Figure 3:**
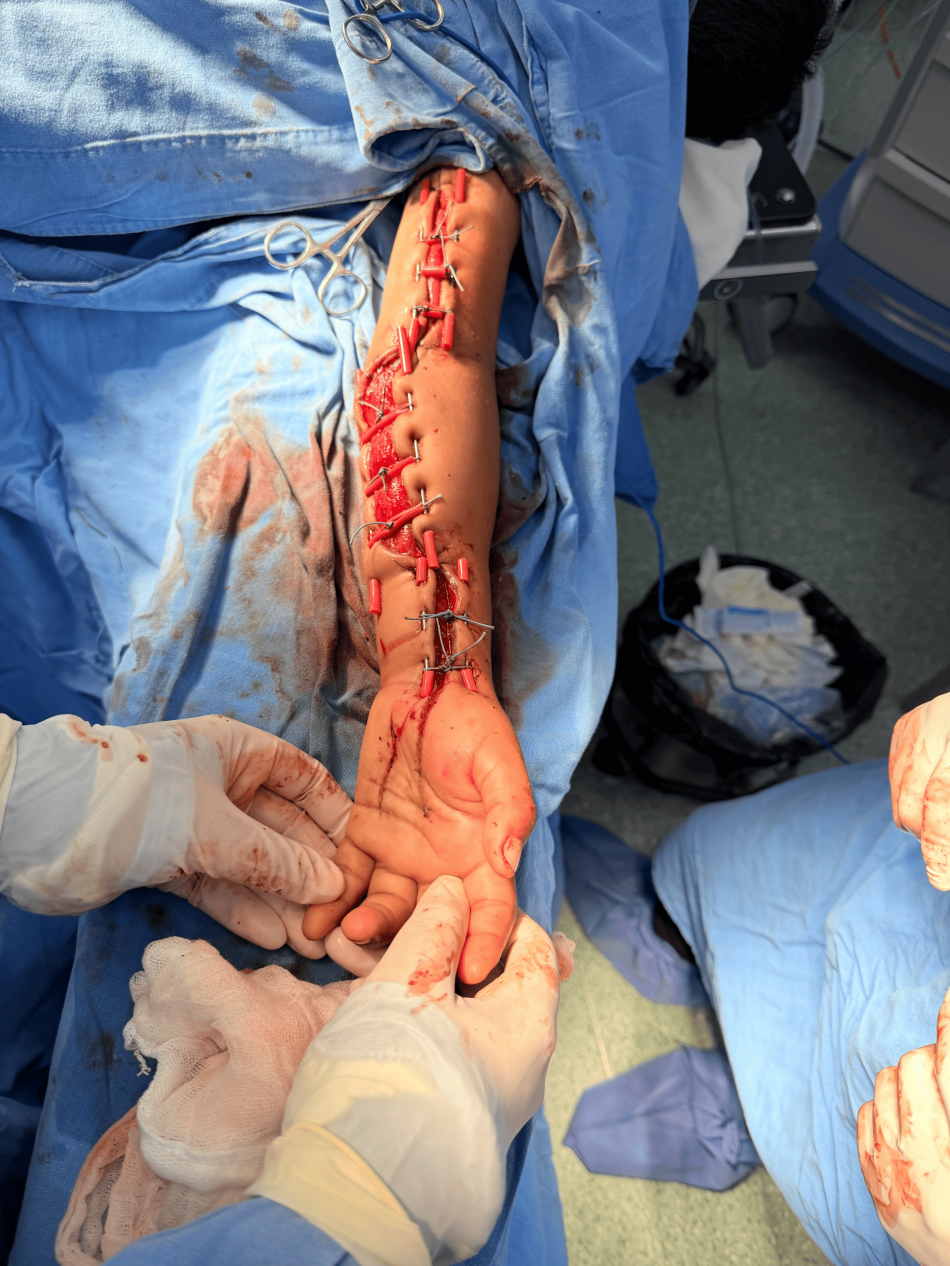
Modified shoelace technique using Kirschner wires Kirschner wires and silicone protectors applied for progressive closure.

The wounds were initially managed open with sterile gauze dressings and nitrofural cream, with dressing changes every 24 hours, as a low-cost protective measure before delayed closure. This approach allowed maintenance of wound coverage, reduction of local contamination, and serial assessment of tissue viability and edema progression. Delayed progressive closure was not initiated earlier because persistent edema initially prevented safe approximation of the wound edges without excessive tension or risk of compromising local perfusion. During this period, no purulence, necrosis, or clinical signs of wound infection were observed, and wound culture results were negative. Seven days later, delayed progressive closure was planned because edema had decreased sufficiently to permit gradual approximation of the wound edges under safe local conditions. At the beginning of progressive closure, the wound remained approximately 40 cm in length, with a maximum width of 8 cm. Six 1.5 mm Kirschner wires were placed along the wound edges at the forearm, wrist, and arm, and polyester 0 sutures were applied in a shoelace configuration. Silicone protectors fashioned from Nelaton catheter segments were placed to reduce localized pressure over the skin. Figure 3 shows the modified shoelace dermatotraction setup using Kirschner wires and silicone protectors.

**Figure 4:**
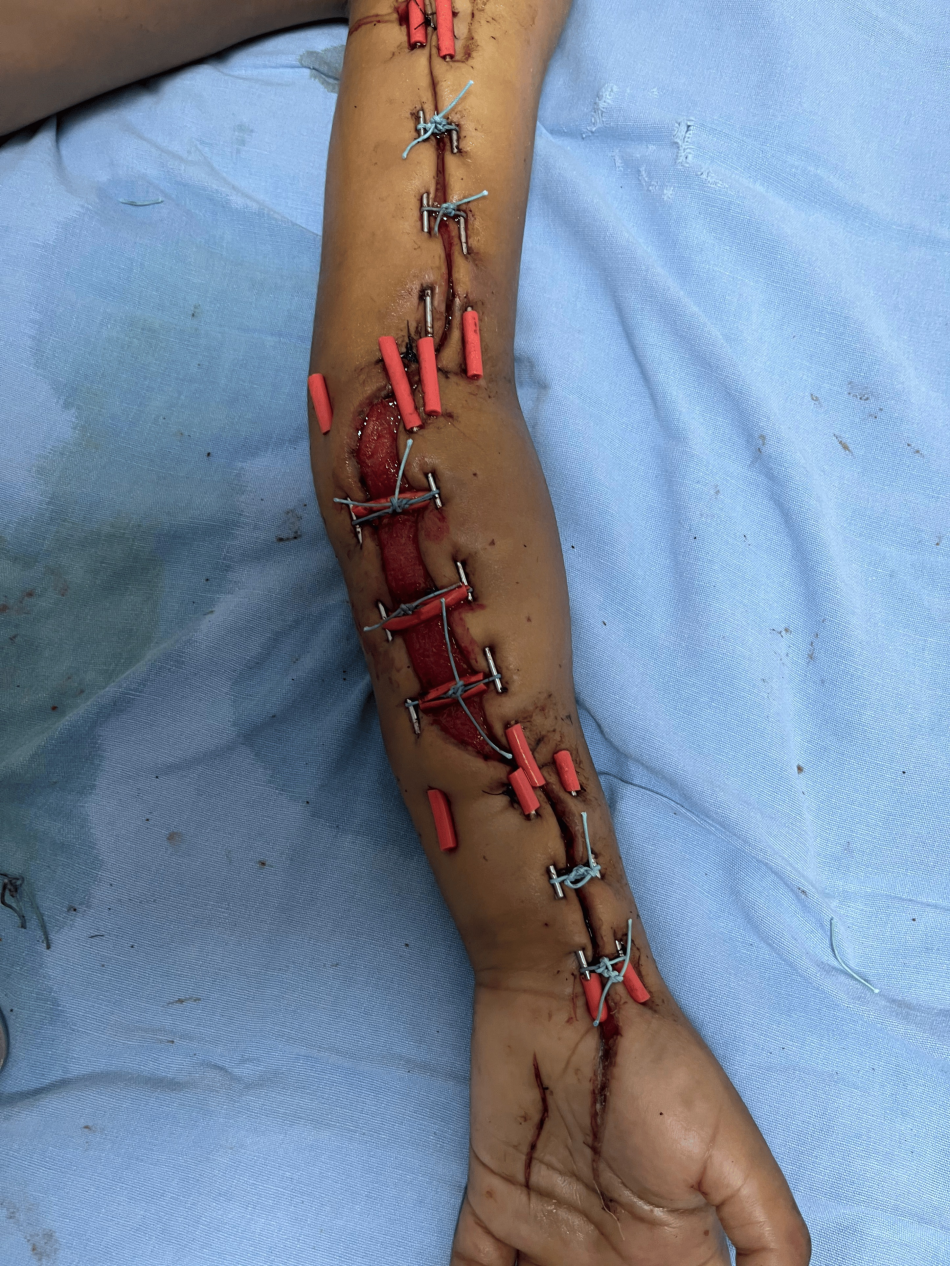
Partial closure at 24 hours Progressive approximation of wound edges without tissue compromise.

Progressive tightening was then performed every 24 hours under sedation. At 24 hours, the maximum wound width had decreased to 6 cm, and pain was rated as 6/10 on the Visual Analog Scale only during manipulation. Figure 4 shows the partial wound approximation achieved after 24 hours of progressive closure.

**Figure 5:**
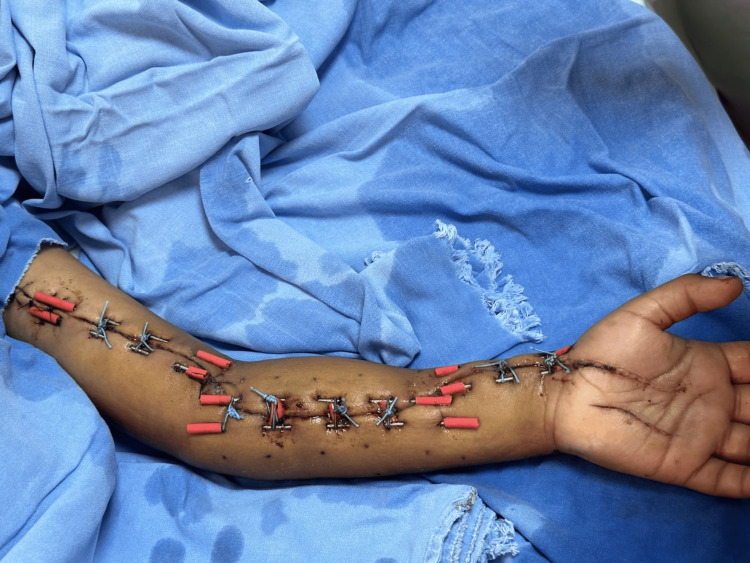
Complete closure at 72 hours Full wound closure achieved without the need for grafting.

Complete closure was obtained at 72 hours, without the need for grafting or negative pressure therapy. Figure 5 shows the wound after complete closure at 72 hours.

**Figure 6:**
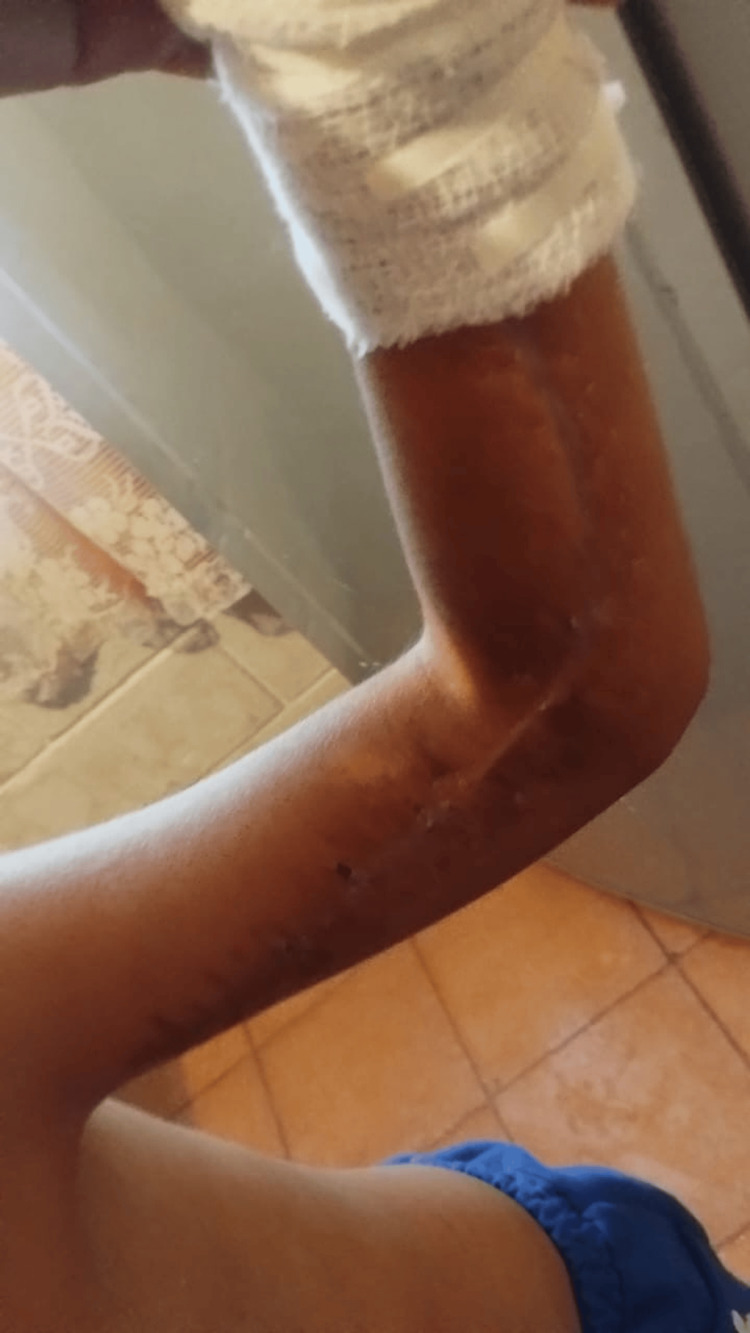
Eight-month postoperative outcome Healed volar forearm scar at eight months of follow-up, with preserved contour and no evidence of contracture.

The Kirschner wires were removed seven days later, and the closure was reinforced with U-stitches. The patient completed 15 days of hospitalization without infection, dehiscence, or necrosis. At eight-month follow-up, he had preserved active and passive range of motion of the wrist and fingers, with strength graded at 4/5. Sensory examination showed preserved sensation in the median, ulnar, and radial nerve distributions of the hand and forearm. Light touch and pinprick sensation were normal over the palmar surface, dorsal hand, thumb, index finger, little finger, and remaining digits, without distal hypoesthesia or dysesthesia. Sensation directly over the scar was limited, consistent with a localized postoperative sensory alteration; however, sensation in the surrounding skin and in the rest of the hand and forearm was normal. The patient returned to school one month after discharge. Rehabilitation included use of a low-cost robotic articulated hand device for 15 minutes every two hours, three sessions per day. Figure 6 shows the late postoperative appearance at eight months of follow-up.

## Discussion

Compartment syndrome secondary to snakebite envenomation is an uncommon but potentially limb-threatening condition [2,3]. Its diagnosis remains challenging, as venom-induced edema may mimic increased compartment pressure, leading to both overdiagnosis and delayed intervention [3]. In the present case, the diagnosis was established clinically based on progressive signs of neurovascular compromise, including pallor, delayed capillary refill, decreased distal temperature, diminished pulses, and paresthesias. Although intracompartmental pressure measurement is recommended when available, its absence should not delay surgical intervention when clinical findings are evident [3,4].

Early administration of antivenom remains the cornerstone of treatment in crotaline envenomation and may reduce local tissue damage [4]. However, in selected patients with clear evidence of compartment syndrome, surgical decompression remains necessary [3,4]. In this case, despite the timely administration of polyvalent antivenom, clinical deterioration justified urgent fasciotomy. Intraoperative findings of marked edema under tension with preserved muscle viability support both the indication and timing of the procedure.

Wound closure following fasciotomy represents a reconstructive challenge. Persistent edema frequently prevents primary closure, and conventional strategies include healing by secondary intention, negative pressure wound therapy, or split-thickness skin grafting [5]. While effective, these approaches may increase costs, prolong hospitalization, and result in less favorable cosmetic and functional outcomes, particularly in pediatric patients [5].

Dermatotraction techniques, such as the shoelace method, have been widely described as effective alternatives for fasciotomy closure [5-7]. These techniques rely on the viscoelastic properties of the skin, particularly mechanical creep and stress relaxation, which allow gradual approximation of wound edges under sustained controlled tension [8,9]. In practical terms, progressive traction produces biological and mechanical deformation of the skin and subcutaneous tissues, permitting delayed closure while reducing the need for grafting when tissue viability is preserved, and edema has sufficiently subsided [8,9].

In the present case, a technical modification of this principle was used, employing Kirschner wires as anchoring points and silicone protectors fashioned from Nelaton catheter segments. Kirschner wires were considered a practical alternative anchoring method because they were readily available in our setting, allowed stable fixation along the wound edges, and facilitated a more uniform distribution of tension during progressive approximation. In addition, their use enabled staged tightening in a simple and reproducible manner without the need for commercial dermatotraction devices. The silicone protectors reduced localized pressure and minimized the risk of skin injury or suture cut-through. This approach may be particularly useful in resource-limited settings where commercial systems are not readily available.

Another relevant aspect in our case was the timing of delayed closure. Dermatotraction was not initiated earlier because persistent edema initially prevented safe approximation of the wound edges without excessive tension or risk of compromising local perfusion. During this interval, the wound was managed with sterile gauze dressings and nitrofural cream, with dressing changes every 24 hours, while tissue viability, edema progression, and signs of infection were serially assessed. No purulence, necrosis, or clinical signs of wound infection were observed, and wound culture results were negative. Therefore, the main reason for initiating progressive closure on postoperative day 7 was the persistence of edema during the early postoperative period rather than infection or tissue deterioration.

Progressive closure was achieved over a 72-hour period without complications, with reduction of the maximum wound width from 8 cm at the start of dermatotraction to 6 cm at 24 hours, 4 cm at 48 hours, and complete closure at 72 hours, without the need for skin grafting or negative pressure wound therapy. Pain during manipulation was moderate, with a Visual Analog Scale score of 6/10. At eight-month follow-up, the patient demonstrated preserved active and passive range of motion, intact sensation in the median, ulnar, and radial nerve distributions except for limited sensation directly over the scar, and a satisfactory cosmetic outcome, supporting the effectiveness of this modified approach.

Additional objective outcome measures, such as grip strength by dynamometry and validated scar assessment scales such as the Vancouver Scar Scale or POSAS, were not systematically recorded. Their inclusion would have strengthened the quantitative assessment of outcomes and the reproducibility of the report.

This case highlights the importance of clinical judgment in the management of snakebite-related compartment syndrome and demonstrates that established dermatotraction techniques can be effectively adapted to available resources while maintaining favorable functional and aesthetic outcomes [5-9]. Further studies are required to evaluate the reproducibility and long-term outcomes of this technical modification.

## Conclusions

Compartment syndrome secondary to snakebite envenomation, although uncommon, requires prompt recognition and timely surgical intervention to prevent irreversible damage. This case demonstrates that clinical assessment remains fundamental when diagnostic resources are limited.

The use of a modified shoelace dermatotraction technique with Kirschner wires and silicone protectors allowed effective and progressive closure of fasciotomy wounds without the need for skin grafting or advanced wound therapy. This approach represents a practical and low-cost alternative, particularly valuable in resource-limited settings.

Favorable functional and aesthetic outcomes at follow-up support the safety and effectiveness of this technique. Further studies are needed to validate its reproducibility and broader applicability.
